# 5-Fraction Re-radiosurgery for Progression Following 8-Fraction Radiosurgery of Brain Metastases From Lung Adenocarcinoma: Importance of Gross Tumor Coverage With Biologically Effective Dose ≥80 Gy and Internal Dose Increase

**DOI:** 10.7759/cureus.42299

**Published:** 2023-07-22

**Authors:** Kazuhiro Ohtakara, Kuniaki Tanahashi, Takeshi Kamomae, Kojiro Suzuki

**Affiliations:** 1 Department of Radiation Oncology, Kainan Hospital Aichi Prefectural Welfare Federation of Agricultural Cooperatives, Yatomi, JPN; 2 Department of Radiology, Aichi Medical University, Nagakute, JPN; 3 Department of Neurosurgery, Gifu Prefectural Tajimi Hospital, Tajimi, JPN; 4 Department of Neurosurgery, Nagoya University Graduate School of Medicine, Nagoya, JPN; 5 Radioisotope Research Center, Nagoya University, Nagoya, JPN; 6 Department of Radiology, Nagoya University Graduate School of Medicine, Nagoya, JPN

**Keywords:** pan-negative, iris collimator, cyberknife, biological effective dose, hypofractionation, lung adenocarcinoma, tumor progression, re-irradiation, stereotactic radiosurgery, brain metastasis

## Abstract

The criteria for indication of salvage stereotactic radiosurgery (SRS) for local progression following multi-fraction (mf) SRS of brain metastases (BMs) remain controversial, along with the optimal planning scheme. Herein, we described a case of BMs from pan-negative lung adenocarcinoma (LAC), in which the two lesions of local progression following initial eight-fraction (8-fr) SRS were re-treated with 5-fr SRS with the biologically effective dose (BED_10_) of ≥80 Gy, based on the linear-quadratic (LQ) formula with an alpha/beta ratio of 10. The re-SRS resulted in the alleviation of symptoms and favorable tumor responses with minimal adverse effects during the 7.3-month follow-up. In the lesions of local progression, the gross tumor volume (GTV) coverage with 49.6 Gy (BED_10_ 80 Gy) was generally insufficient, and the GTV dose wes relatively homogeneous with ≥87% isodose covering. In contrast, the 5-fr re-SRS was performed with sufficient GTV coverage with ≤68% isodose of 43 Gy (BED_10_ 80 Gy). Taken together, sufficient GTV coverage with a BED_10_ of ≥80 Gy and steep dose increase inside the GTV boundary, that is, extremely inhomogeneous GTV dose, are important in 8-fr SRS for ensuring excellent local control of BMs from pan-negative LAC. For local progression following mfSRS that does not fulfill both criteria, re-SRS with the above planning scheme can be an efficacious and safe treatment option for at least six months, especially in cases in which the prior SRS was performed with a dose/fractionation under adequate consideration of brain tolerance. The BED_10_ seems to be the most suitable for estimating the anti-tumor efficacies of SRS doses in 3-8 fr, similar to that of a single fraction of 24 Gy.

## Introduction

Stereotactic radiosurgery (SRS) is an essential local treatment option for non-miliary and non-disseminated brain metastases (BMs) from non-small cell lung cancer (NSCLC); however, its dose gradient outside and inside the tumor margin and dose fractionation remain highly variable among institutions [1,2]. In response to the target-volume limitation of single-fraction SRS regarding efficacy and safety, three- to five-fraction (3-5 fr) SRS is increasingly applied to ≥1.5-2 cm BM, for which 27-30 Gy in 3 fr and 30-35 Gy in 5 fr are common schedules [1,3,4]. The corresponding biologically effective doses (BED) that are based on the standard linear-quadratic formula with an alpha/beta ratio of 10 (BED_10_) are approximately 50-60 Gy [5]. However, approximately 20% of local tumor progression generally occurs within one year when these dose fractionation schemes are used [1]. Meanwhile, Matsuyama et al. stated that 2-10 fr SRS with the BED_10_ of ≥80 Gy prescribed to 90% isodose surface (IDS) covering 1-2 mm outside the gross tumor volume (GTV) boundary can improve local control of BMs from NSCLC; however, the one-year local control rate for >2 cm BMs was ≤85% [6]. Additionally, adverse radiation effects (AREs) generally increase significantly when the irradiated isodose volumes, including GTVs, receiving ≥20 Gy in 3 fr or ≥24 Gy in 5 fr exceed >20 cm^3^ [3,7]. Therefore, volume effects should also be considered, even in multi-fraction (mf) SRS. In cases of more than four BMs and/or brainstem involvement, the prescription dose may be compromised, considering the increased risk of AREs. Furthermore, for radiographic local progression following a nadir response after SRS, the criteria for determining whether tumor regrowth or ARE predominates and re-SRS indication remain controversial, along with the optimal dose fractionation scheme and dose distribution for re-SRS [8,9].

Herein, we present a case of BMs from lung adenocarcinoma (LAC) harboring no common driver genetic alterations that were initially treated with 8-fr SRS without subsequent anti-cancer pharmacotherapy [10]. Despite the GTV coverage with the BED_10_ of 80 Gy, four of the six BMs developed local progression. Two of the four lesions were re-irradiated with 5-fr SRS adopting sufficient GTV coverage with the BED_10_ of 80 Gy and a steep internal dose increase (extremely inhomogeneous GTV dose). Through a description of the clinical course along with detailed analyses of the planning parameters for the initial and re-SRS, we discuss several relevant issues as follows: the optimal dose distribution of 8-fr SRS for ensuring long-term sustained local control, criteria for determining whether tumor regrowth or ARE predominates in local progression following SRS, indication and validity of re-SRS with a BED_10_ of 80 Gy, and optimal BED formula for estimating anti-tumor efficacy in 3-8 fr SRS.

Part of this study was previously presented at the 30^th^ Annual Meeting of the Japanese Society for Stereotactic Radiosurgery, held online on June 11 to July 8, 2021.

## Case presentation

A 70-year-old, right-handed male who was an ex-smoker presented with unsteadiness and mild left-sided hemiparesis. Ten months earlier, the patient underwent left upper lobectomy with regional lymph node dissection for lung adenocarcinoma (LAC) detected by screening. Pathological examination revealed mediastinal lymph node metastases, negative for major genetic alterations, and programmed cell death ligand-1 (PD-L1) expression of 5-10%. The patient received four courses of adjuvant chemotherapy consisting of cisplatin and vinorelbine until four months before the onset of BMs. Magnetic resonance imaging (MRI) revealed six lesions compatible with BMs, and systemic examination revealed no obvious extracranial recurrences or metastases. The Lung-molGPA score proposed by Sperduto et al. was 1.5 (Karnofsky Performance Score: 80%, extracranial metastasis: absent) [11]. The neurological symptoms were mainly attributed to the two BMs located in the right thalamo-mesencephalon and the right superior parietal lobule (near the arcus parietooccipitalis). These six BMs were initially treated with SRS alone using CyberKnife (CK) M6 (Sunnyvale, CA: Accuray Inc.) with 6 MV x-rays [12].

**Figure 1:**
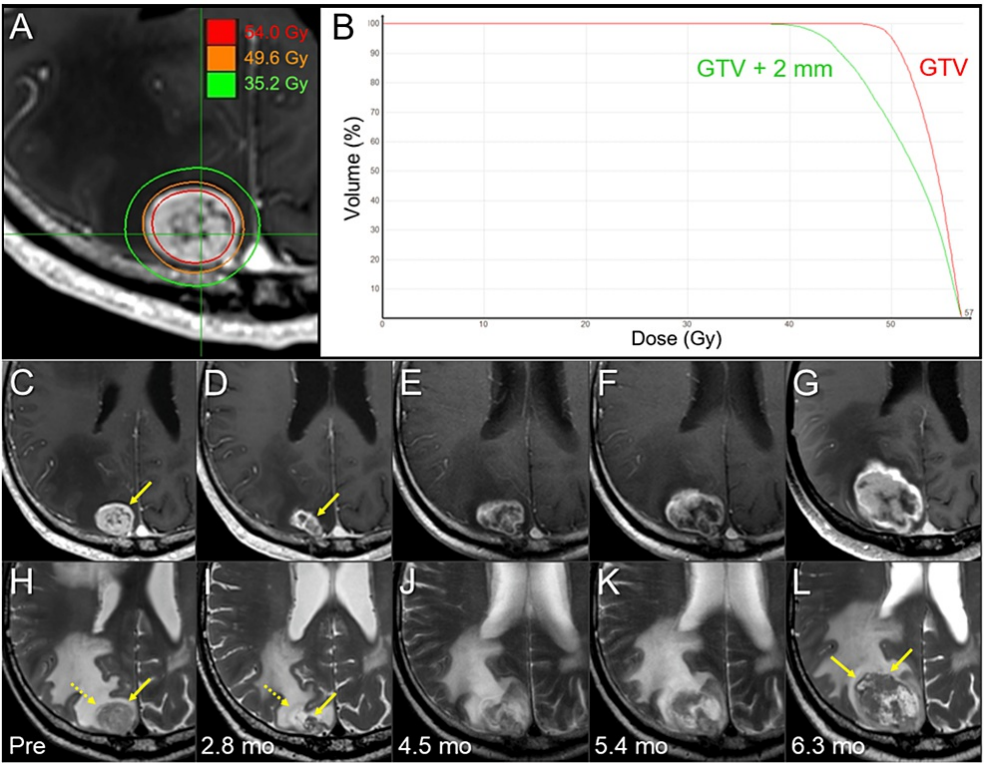
Dose distribution, dose-volume histograms, and magnetic resonance images from before 8-fr stereotactic radiosurgery to before salvage surgery in the right parietal lesion. The images show an axial dose distribution (A); dose-volume histograms (DVHs) (B); axial contrast-enhanced (CE) T1-weighted images (WIs) (C-G); axial T2-WIs (H-L); five days before the initiation of stereotactic radiosurgery (SRS) (C, H); at 2.8 months (mo) after SRS initiation (D, I); at 4.5 months (E, J); at 5.4 months (F, K); and at 6.3 months (G, L). (A) Three representative isodose lines are superimposed onto an axial CE-T1-WI. (B) The gross tumor volume (GTV) +2 mm structure was generated by adding an isotropic 2-mm margin to the gross tumor volume (GTV) periphery. (C-L) These images are shown at the same magnification and coordinates under co-registration and fusion. (C, H) The mainly solid mass lesion (arrows in C, D) associated with perilesional edema in the right parietal lobe. (D, I) At 2.8 months after SRS, the lesion shrank markedly; however, it left a distinct solid component (arrows in D, I) with T1/T2 matching. The single-layered structure (dashed arrow in I) was observed around the enhancing lesion, which may be relevant to the partly cystic component (dashed arrow in H) before SRS. (E-G, J-L) After the maximum response at 2.8 months, the enhancing lesion obviously increased in size from 4.5 months to 6.3 months, and the clear mass also became noticeable on the T2-WIs (arrows in L).

**Figure 2:**
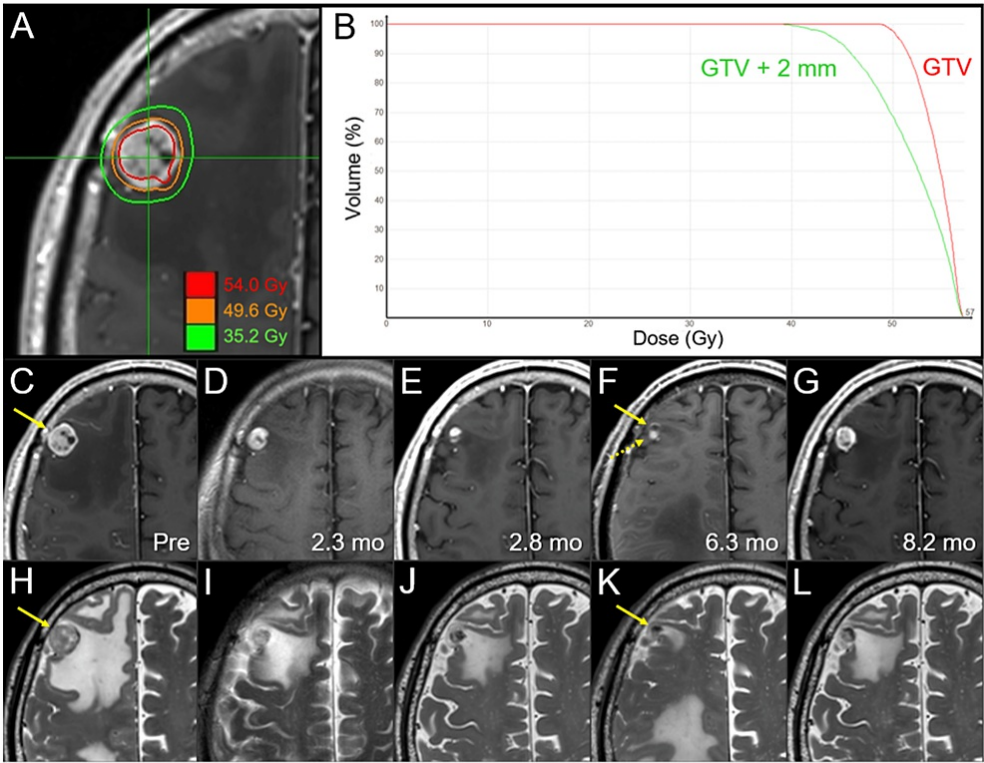
Dose distribution, dose-volume histograms, and magnetic resonance images from before 8-fr stereotactic radiosurgery (SRS) to before salvage 5-fr SRS in the right frontal lesion. The images show dose distribution (A); DVHs (B); axial CE-T1-WIs (C-G); axial T2-WIs (H-L); five days before stereotactic radiosurgery (SRS) (C, H); at 2.3 months (mo) after SRS (D, I); at 2.8 months (E, J); at 6.3 months (F, K); and at 8.2 months (G, L). (C-L) These images are shown at the same magnification and coordinates under co-registration and fusion. (C, H) The solid mass lesion (arrows in C, D) associated with perilesional edema in the right frontal lobe. (C-F, H-K) The lesion gradually shrank, and the surrounding edema decreased over 6.3 months after SRS. At the maximum response at 6.3 months, the peripherally enhancing mass (arrow in F) and the solid-enhancing one (dashed arrow in F) were adjacent to each other, in which the former was low intensity on the T2-WI (arrow in K). (G, L) After the nadir response at 6.3 months, both the enhancing lesion and the visible mass on the T2-WI obviously enlarged, along with increased perilesional edema at 8.2 months. DVHs: dose-volume histograms; CE: contrast-enhanced; WI: weighted image; GTV: gross tumor volume

**Figure 3:**
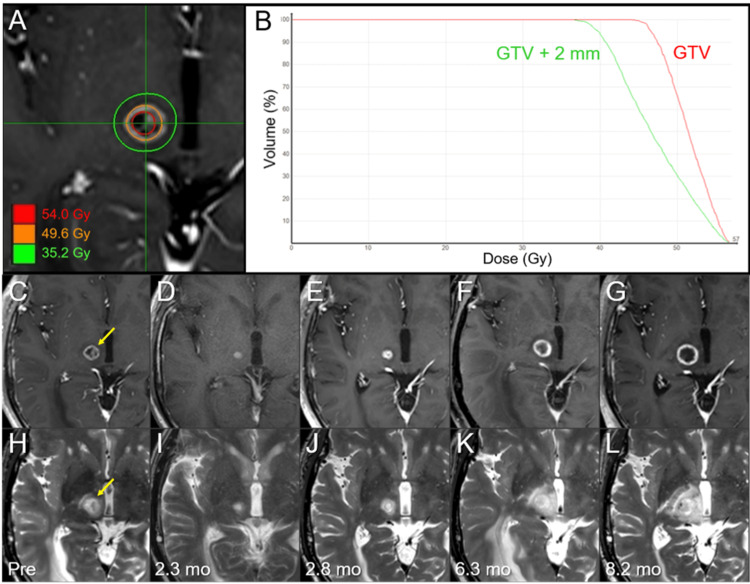
Dose distribution, dose-volume histograms, and magnetic resonance images from before 8-fr stereotactic radiosurgery (SRS) to before salvage 5-fr SRS in the right thalamo-mesencephalic lesion. The images show dose distribution (A); DVHs (B); axial CE-T1-WIs (C-G); axial T2-WIs (H-L); five days before SRS (C, H); at 2.3 months (mo) after SRS (D, I); at 2.8 months (E, J); at 6.3 months (F, K); and at 8.2 months (G, L). (C-L) These images are shown at the same magnification and coordinates under co-registration and fusion. (C, H) The heterogeneously enhancing lesion (arrow in C) in the right thalamo-mesencephalic junction shows the indistinct boundary concomitant with the perilesional high-intensity area on T2-WI (arrow in H). (D, I) The lesion remarkably shrank at 2.3 months after SRS. (E-G, J-L) The lesion obviously enlarged with T1/T2 matching from 2.8 months to 8.2 months, along with increased perilesional edema. At 6.3 months, ventral displacement of the lesion was observed, which was attributed to the increased mass effect due to the progression of the right parietal lesion. DVHs: dose-volume histograms; CE: contrast-enhanced; WI: weighted image; GTV: gross tumor volume

Since 2018, to achieve longer-lasting tumor shrinkage and enhance long-term safety for BMs up to approximately 5 cm, we shifted to the dose prescription regimen of ensuring GTV coverage with BED_10_ of ≥80 Gy along with versatile and flexible dose fractions ranging from 3 to 15 [5,8,12-14]. Given the largest lesion volume, each localization (brainstem involvement), and the distance between lesions, ≥5 fr was deemed suitable for SRS of the present case [3,7]. Finally, 49.6 Gy in 8 fr was adopted for the GTV prescription dose to ensure long-term safety, considering the effects of dose interference due to the simultaneous irradiation of six lesions and the predicted prognosis of isolated central nervous system (CNS) metastases. The GTVs were defined as enhancing lesions that were nearly consistent with the visible masses on T2-weighted images (T1/T2 matching) [8,12]. The optimization algorithm for SRS planning was Sequential (Sunnyvale, CA: Accuray Inc.), built into the dedicated planning system Precision (Sunnyvale, CA: Accuray Inc.). Simultaneous and comprehensive optimization with a single path (plan) using 178 beams from 98 nodes was adopted to efficiently irradiate the six lesions in a short time, for which a regular dodecagon-shaped variable-sized collimator Iris (Sunnyvale, CA: Accuray Inc.) was leveraged [12]. The estimated treatment time (EST) was 39 minutes per fraction (mean 6.5 minutes per lesion). The dose distributions, dose-volume histograms, and MRIs before and after SRS for the three large lesions are shown in Figures 1A-1L, 2A-2L, 3A-3L.

**Figure 4:**
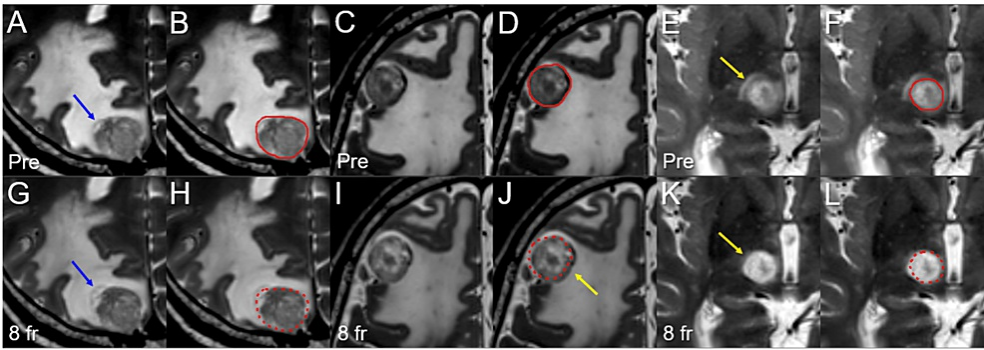
T2-weighted images before and at the completion of 8-fr stereotactic radiosurgery. The images show axial T2-WIs (A-L); five days before the initiation of SRS (A-F); at the completion of 8-fr SRS (G-L); the right parietal lesion (A, B, G, H); the right frontal lesion (C, D, I, J); the right thalamo-mesencephalic lesion (E, F, K, L); the GTVs (solid lines) contoured on the T2-WIs before SRS (B, D, F); and the pre-SRS GTV contours (dashed lines) superimposed onto the T2-WIs at the completion of SRS (H, J, L). (A-L): All images are shown at the same magnification and coordinates under co-registration and fusion. (A, B, G, H) The partly cystic component in the right parietal lesion (blue arrow in A) showed expansion beyond the pre-SRS GTV contour (blue arrow in G) at the completion of SRS. (C, D, I, J) At the completion of SRS, dorsomedial displacement of the GTV (yellow arrow in J) was observed, along with the increased GTV intensity. (E, F, K, L) The perilesional high-intensity area (yellow arrow in E) almost disappeared (yellow arrow in K), and the lesional intensity itself increased at the completion of SRS. WIs: weighted images; SRS: stereotactic radiosurgery; 8-fr: eight-fraction; GTVs: gross tumor volumes

The planning parameters for the six lesions are included in Tables 1, 2. Although ≥98% GTV coverage with a BED10 of 80 Gy was the principle, the GTV coverage with 49.6 Gy in 8 fr was partially compromised as <98%, especially for the thalamo-mesencephalic lesion, considering the non-oligo BMs and the future high probability of new lesions in other intracranial sites. In addition, by selecting larger collimator sizes for Iris, shortening the irradiation time was prioritized over GTV coverage with ≤80% IDS. This was also performed before the installation of CK-VOLO (Sunnyvale, CA: Accuray Inc.) for the optimization algorithm [12]. Accordingly, the GTV doses were homogeneous, and the dose gradients outside the GTV boundary were gradual [2,15]. Regarding the inter-fractional tumor changes and/or displacement, no obvious shrinkage was observed, although enlargement of the partially cystic component and medial displacement of the tumor were observed in one case each (Figures 4A-4L) [8,12,16].

**Table 1:** Tumor characteristics and planning parameters for initial 8-fr and salvage 5-fr stereotactic radiosurgery for large or critical brain metastases. *The BED_10_ for the absolute doses of 54 Gy in 8 fr and 50 Gy in 5 fr are 90.5 Gy and 100.0 Gy, respectively. **The %IDSs for 49.6 Gy in 8 fr and 43.0 Gy in 5 fr (BED_10_ 80 Gy), relative to the D_max_. The% IDSs for 8 fr are ≥87%, whereas those for 5 fr are ≤67.5%. ***The BED_10_ for absolute doses of the D_min_ of each GTV + 2 mm structure. SRS: stereotactic radiosurgery; fr: fraction; Rt: right; GTV: gross tumor volume; D_max_: maximum dose; BED_10_: a biologically effective dose based on the linear-quadratic formula with an alpha/beta ratio of 10; IDS: isodose surface; D_min_: minimum dose

SRS		Initial SRS (8 fr)			Re-SRS (5 fr)	
Tumor location		Rt parietal surface	Rt frontal surface	Rt thalamo-mesencephalic	Rt frontal surface	Rt thalamo-mesencephalic
GTV	Volume	5.30 cm^3^	2.87 cm^3^	0.76 cm^3^	1.94 cm^3^	1.80 cm^3^
	D_max_	57.0 Gy	57.0 Gy	56.8 Gy	63.7 Gy	68.6 Gy
	Internal dose*: dose (coverage)	54.0 Gy (56.9%)	54.0 Gy (61.1%)	54.0 Gy (22.3%)	50.0 Gy (71.0%)	50.0 Gy (74.5%)
	BED_10_ 80 Gy: dose (coverage)	49.6 Gy (96.6%)	49.6 Gy (98.4%)	49.6 Gy (70.0%)	43.0 Gy (98.8%)	43.0 Gy (99.8%)
	% IDS**	87.0%	87.0%	87.3%	67.5%	62.7%
	D_min_	44.9 Gy	47.7 Gy	43.1 Gy	39.8 Gy	42.0 Gy
GTV + 2 mm	BED_10_ 60 Gy: dose (coverage)	40 Gy (99.4%)	40 Gy (99.4%)	40 Gy (93.3%)	35.3 Gy (92.7%)	35.3 Gy (91.8%)
	D_min_	35.7 Gy	37.3 Gy	36.3 Gy	27.4 Gy	30.7 Gy
	BED_10_***	51.6 Gy	54.7 Gy	52.8 Gy	42.4 Gy	49.6 Gy
Iris collimator size		25, 30 mm	20, 25 mm	20 mm	12.5, 15, 20, 25 mm	15, 20, 25 mm
Target boundary distance		-5 mm	-5 mm	-1 mm	-2 mm	-3 mm

**Table 2:** Tumor characteristics and planning parameters for muti-fraction stereotactic radiosurgery of small brain metastases with the gross tumor volume of <0.7 cc. *For 3-fr SRS of the six BMs, the ranges of variables are shown. **The %IDS of the GTV D_min_ relative to the GTV D_max_ (100%). ***The BED_10_ for the absolute doses of each GTV D_min_. SRS: stereotactic radiosurgery; fr: fraction; GTV: gross tumor volume; Rt: right; Lt: left; D_max_: maximum dose; D_min_: minimum dose; IDS: isodose surface; BED_10_: a biologically effective dose based on the linear-quadratic formula with an alpha/beta ratio of 10; mo: months; PD: progressive disease; CR: complete remission; nCR: nearly CR; BMs: brain metastases

SRS		1st (8 fr)			2nd (3 fr)*	3rd (5 fr)	
Location		Rt frontal (pre-central gyrus)	Rt frontal deep (paraventricular)	Lt cerebellar deep	(Various)	Rt parietal surface	Lt frontal surface
GTV	Volume	0.13 cm^3^	0.05 cm^3^	0.07 cm^3^	0.01-0.25 cm^3^	0.40 cm^3^	0.66 cm^3^
	D_max_ (D_0.001 cc_)	53.6 Gy	53.4 Gy	56.9 Gy	36.1-39.6 Gy	58.0 Gy	57.9 Gy
	D_min_	48.9 Gy	49.0 Gy	49.4 Gy	35.0-35.2 Gy	43.3 Gy	44.3 Gy
	%IDS**	91.2%	91.8%	86.8%	88.9-97.5%	74.7%	76.5%
	BED_10_	78.8 Gy	79.0 Gy	79.9 Gy	75.8-76.5 Gy	80.8 Gy	83.6 Gy
GTV + 2 mm	D_min_	38.1 Gy	38.1 Gy	36.9 Gy	28.4-31.1 Gy	30.4 Gy	31.5 Gy
	BED_10_***	56.3 Gy	56.3 Gy	53.9 Gy	55.3-63.3 Gy	48.9 Gy	51.4 Gy
Imaging follow-up		15.7 mo	15.7 mo	15.7 mo	12.7 mo	7.3 mo	7.3 mo
Final response		PD	CR	nCR	CR	CR	CR

Owing to anti-brain edema medication, followed by tumor shrinkage, the patient’s neurological symptoms improved during SRS and almost disappeared within one month. After SRS, the patient was followed up without subsequent anti-cancer pharmacotherapy, considering isolated CNS failure. The anti-cancer treatments administered after the initial SRS are summarized in Table 3.

**Table 3:** Summary of anti-cancer treatments after initial 8-fr stereotactic radiosurgery. mo: months; SRS: stereotactic radiosurgery; fr: fraction; BMs: brain metastases; mets: metastases; CBDCA: carboplatin; PTX: paclitaxel; Bev: bevacizumab; RT: radiotherapy

Time	Anti-cancer treatments
3.0 mo	2nd SRS (3 fr) for 6 new BMs
6.3 mo	Salvage surgery for regrowth of BM treated with initial SRS
8.4 mo	3rd SRS (5 fr) for 2 new BMs, limited local recurrences in the post-resection cavity, and salvage of the 2 regrowing BMs after initial SRS
8.6 mo	Detection of spinal mets
8.6 mo	CBDCA + PTX + Bev + atezolizumab (3 course)
11.0 mo	4th SRS (5 fr) for limited meningeal dissemination near the post-resection cavity
12.0 mo	Transition to palliative care
15.0 mo	Palliative RT for spinal mets
18.8 mo	Deceased

Two months after SRS, MRI revealed six new BMs and partial responses (PRs) in the initially treated lesions. Upon retrospective review, five of the six new lesions were faintly visible on the pre-SRS MRI; thus, the present case had at least 11 BMs before treatment. Three months after the initial SRS, the six new BMs were simultaneously treated with a second 3-fr SRS using CK with 86 beams from 63 nodes, in which the basic GTV marginal doses were 36.3 Gy (BED_10_ 80 Gy) (Table 2). The EST was 33 minutes per fraction (mean 5.5 minutes per lesion).

**Figure 5:**
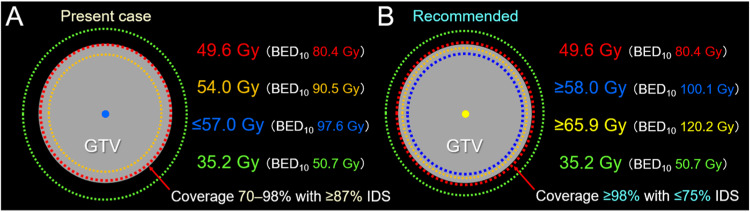
Difference in treatment planning for 8-fr stereotactic radiosurgery of a brain metastasis. The schemas show a GTV covered by the IDS of a prescribed dose of 49.6 Gy, and the dose gradient inside and outside the GTV boundary (A, B); the dose distribution in the present case of local progression following SRS (A); and the recommended one (B). (A) The D≤98% of a GTV is covered by 49.6 Gy with ≥87% IDS, and the maximum dose is ≤57 Gy (BED_10_ <100 Gy). The fairly homogeneous GTV dose is associated with the gradual, not steep, dose gradient outside the GTV boundary: over-coverage with 35.2 Gy. (B) The D≥98% of GTV should be covered by ≤75% IDS, and the maximum dose should be ≥65.9 Gy (BED_10_ ≥120 Gy). Thus, concentrically-laminated steep dose increase just inside the GTV boundary is important for achieving early and adequate tumor shrinkage followed by complete tumor necrosis. The extremely inhomogeneous GTV dose leads to the steep dose gradient outside the GTV boundary - moderately steep dose spillage to 35.2 Gy. GTV: gross tumor volume; IDS: isodose surface; SRS: stereotactic radiosurgery; BED_10_: a biologically effective dose based on the linear-quadratic formula with an alpha/beta ratio of 10

Thereafter, the size of the right parietal and thalamo-mesencephalic lesions gradually increased, and the patient experienced weakness and numbness in the left upper and lower extremities (Figures 1A-1L, 3A-3L). Therefore, the right parietal lesion was completely removed 6.3 months after the initial SRS, using neuronavigation and intraoperative MRI under general anesthesia. The dominance of viable cancers was verified by pathological examination. Postoperative recovery was uneventful; however, the patient’s numbness and weakness gradually progressed. MRI obtained 8.2 months after the initial SRS revealed further enlargement of the right thalamo-mesencephalic lesion (Figures 3A-3L and Table 1), obvious enlargement of the right frontal lesion (Figures 2A-2L and Table 1), two limited locally recurrent lesions in the post-resection cavity (image not shown), and two new lesions (Table 2). The dose distribution design for the initial 8-fr SRS, which mostly resulted in PRs followed by regrowth, is shown in Figures 5A, 5B.

**Figure 6:**
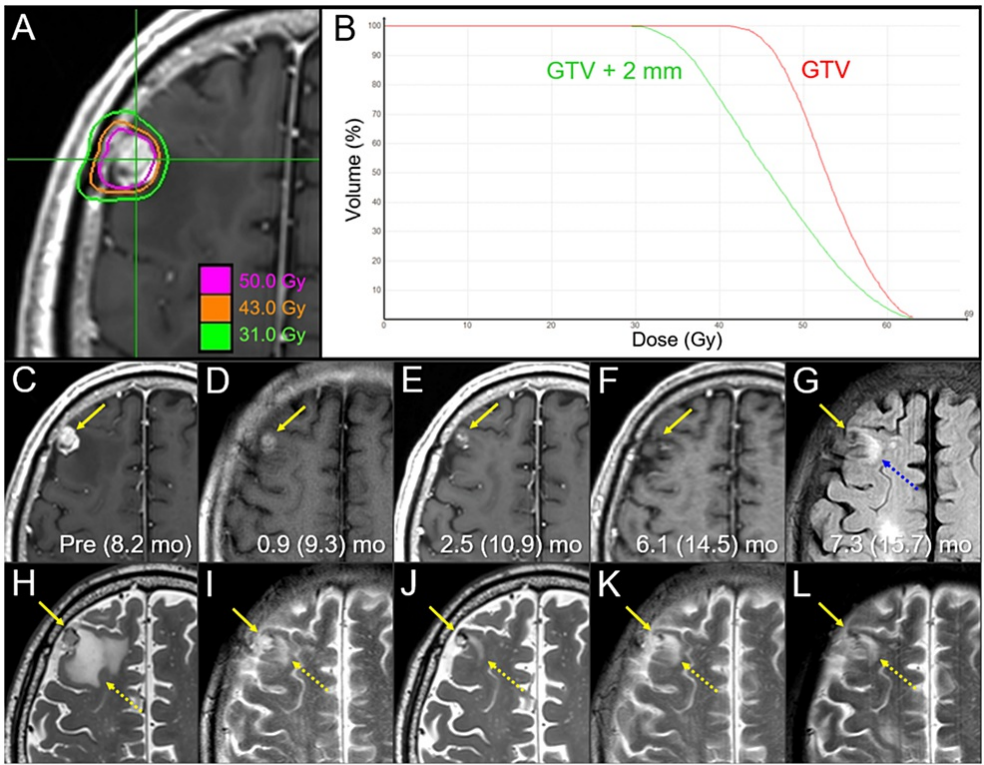
Dose distribution, dose-volume histograms, and magnetic resonance images before and after salvage 5-fr stereotactic re-radiosurgery in the right frontal lesion. The images show dose distribution (A); DVHs (B); axial CE-T1-WIs (C-F); fluid-attenuated inversion recovery (FLAIR) image (G); axial T2-WIs (H-L); at 8.2 months (mo) after the initial 8-fr SRS (six days before the salvage 5-fr re-SRS) (C, H); at 0.9 months after the re-SRS (9.3 months after the initial SRS) (D, I); at 2.5 (10.9) months (E, J); at 6.1 (14.5) months (F, K); and at 7.3 (15.7) months (G, L). (C-L) These images are at the same magnification and coordinates under co-registration and fusion. (C, H) The enhancing area of the lesion (arrow in C) exceeded the visible mass on T2-WI (arrow in H), which was associated with perilesional edema (dashed arrow in H). (D, E, I, J) From 0.9 months to 2.5 months after the re-SRS, the enhancing lesion markedly shrank, along with attenuation of the enhancing effect (arrows in D, E), the internal intensity of the visible mass on T2-WI increased (arrows in I, J), and the perilesional edema markedly decreased (yellow dashed arrows in I, J). (F, K) At 6.1 months, both the enhancing lesion (arrow in F) and the visible mass on T2-WI (arrow in K) further became obscure; however, the perilesional edema increased (yellow dashed arrow in K). (G, L) At 7.3 months, the lesion remained regressed (arrows in G, L) without expansion of the perilesional edema (yellow and blue dashed arrows in G and L, respectively). DVHs: dose-volume histograms; CE: contrast-enhanced; WI: weighted image; fr: fraction; SRS: stereotactic radiosurgery; GTV: gross tumor volume

**Figure 7:**
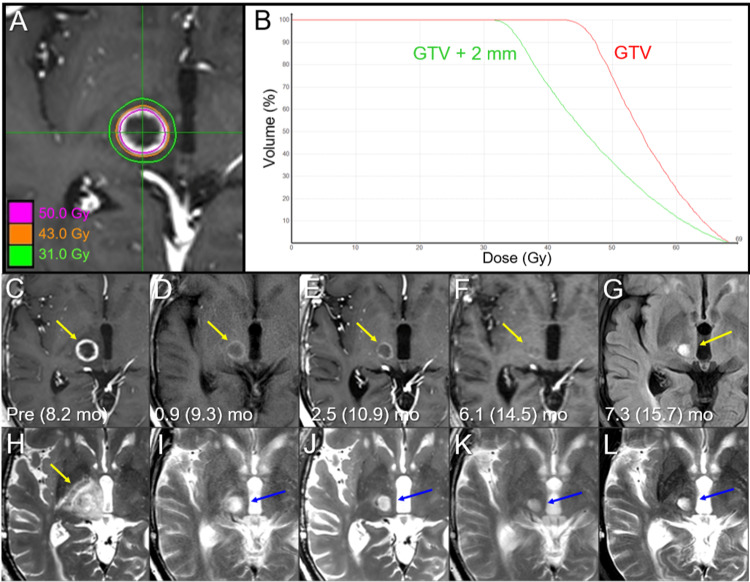
Dose distribution, dose-volume histograms, and magnetic resonance images before and after salvage 5-fr stereotactic re-radiosurgery in the right thalamo-mesencephalic lesion. The images show dose distribution (A); DVHs (B); axial CE-T1-WIs (C-F); FLAIR image (G); axial T2-WIs (H-L); at 8.2 months (mo) after the initial 8-fr SRS (at six days before the initiation of salvage 5-fr re-SRS) (C, H); at 0.9 months after the re-SRS (9.3 months after the initial SRS) (D, I); at 2.5 (10.9) months (E, J); at 6.1 (14.5) months (F, K); and at 7.3 (15.7) months (G, L). (C-L) These images are shown at the same magnification and coordinates under co-registration and fusion. (C, H) The ring-like enhancing lesion (arrow in C) was visualized as the ill-demarcated mass on T2-WI (arrow in H), and the perilesional edema extended to almost the entire ipsilateral thalamus. (D, E, I, J) From 0.9 months to 2.5 months after the re-SRS, the enhancing lesion shrank (arrows in D, E), the enhancing effect was remarkably attenuated (arrows in D, E), and the perilesional edema almost disappeared, along with extrication of the mass effect (blue arrows in I, J). (F, K) At 6.1 months, the enhancing effect almost disappeared (arrow in F), and the mass lesion visible on T2-WI further shrank (blue arrow in K). (G, L) At 7.3 months, the lesion remained regressed and; however, became somewhat more noticeable as a cavitary change (yellow and blue arrows in G and L, respectively). DVHs: dose-volume histograms; CE: contrast-enhanced; WI: weighted image; FLAIR: fluid-attenuated inversion recovery; fr: fraction; SRS: stereotactic radiosurgery; GTV: gross tumor volume

**Figure 8:**
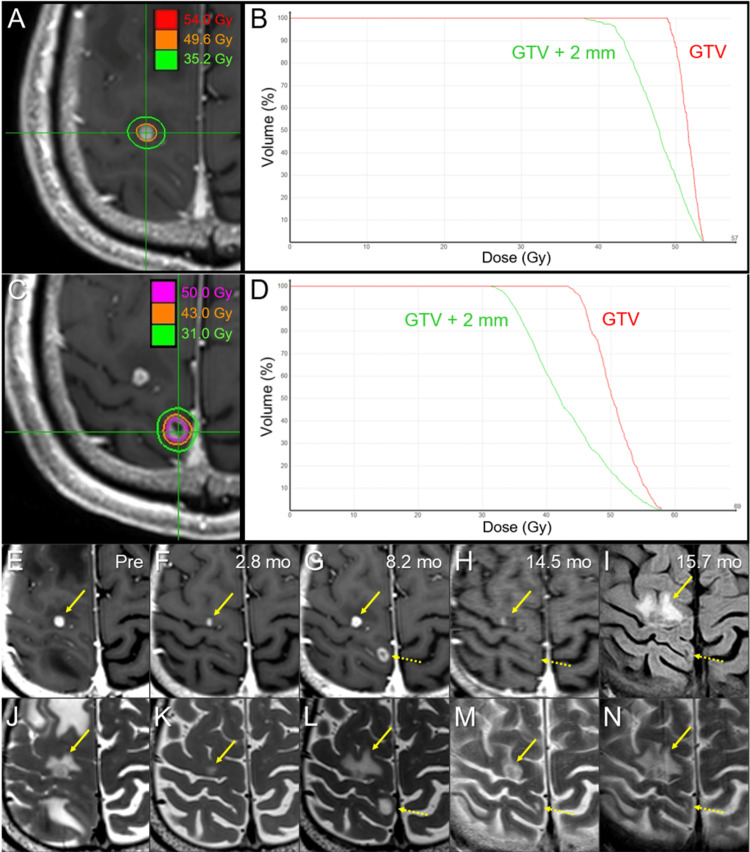
Dose distributions, dose-volume histograms, and magnetic resonance images before and after 8- and 5-fr stereotactic radiosurgery for the right frontal and parietal lesions, respectively. The images show dose distributions (A, C); DVHs (B, D); axial CE-T1-WIs (E-H); axial FLAIR image (I); axial T2-WIs (J-N); the right frontal lesion (A, B); the right parietal lesion (C, D); five days before the 8-fr SRS (E, J); at 2.8 months (mo) after the 8-fr SRS (F, K); at 8.2 months (before the 5-fr SRS) (E, J); at 14.5 months (6.1 months after the 5-fr SRS) (F, K); and at 15.7 (7.3) months (G, L). (C-L) These images are shown at the same magnification and coordinates under co-registration and fusion. (E, J) The solid enhancing lesion (arrow in E) associated with perilesional edema (arrow in J) in the right frontal lobe. (F, K) At 2.8 months after the 8-fr SRS, the lesion shrank obviously (arrow in F), and the perilesional edema almost disappeared (arrow in K). (G, L) At 8.2 months, the lesion obviously enlarged (arrow in G), and the perilesional edema expanded (arrow in L) more extensively before SRS. The new lesion (dashed arrows in G, L) appeared in the right parietal lobe. (H, M) The enhancing effect of the frontal lesion was attenuated (arrow in H), and the perilesional edema decreased (arrow in M). However, the visible mass on T2-WI enlarged (arrow in M), suggesting pseudo-response to chemoimmunotherapy. The parietal lesion shrank markedly, leaving only the cavitary scar (dashed arrows in H, M). (I, N) In the frontal lesion, the visible mass persisted, and the perilesional edema further expanded (arrows in I, N). The parietal lesion remained in nearly complete remission (dashed arrows in I, N). DVHs: dose-volume histograms; CE: contrast-enhanced; WI: weighted image; FLAIR: fluid-attenuated inversion recovery; fr: fraction; SRS: stereotactic radiosurgery; GTV: gross tumor volume

Given the sufficient coverage of 35.2-40 Gy (BED_10_ 50-60 Gy), insufficiency of both the marginal and internal doses of the GTVs was deemed the main cause of PRs and subsequent inevitable regrowth (Figure 5A, 5B). Considering the local progression after initial SRS with 49.6 Gy covering 98.4% of the GTV in the right frontal lesion, we determined the necessity of a higher therapeutic intensity to make re-irradiation effective (Figures 2A-2L). Specifically, sufficient GTV coverage with the BED_10_ of 80 Gy, sufficient increase in the GTV internal dose, steeper dose gradient outside the GTV boundary, and smaller number of fractions were deemed appropriate for re-SRS. A total of six lesions, including the two lesions of local progression following initial SRS, were treated with a third SRS using the GTV marginal dose of 43 Gy in 5 fr using CK with 192 beams from 78 nodes (Figures 6A-6L, 7A-7L, 8A-8N, and Tables 1, 2).

The EST was 45 minutes per fraction (mean 7.5 minutes per lesion). After completion of the third SRS, systemic re-examination revealed metastases to the thoracic spine. Chemoimmunotherapy consisting of carboplatin, paclitaxel, bevacizumab, and atezolizumab (ABCP) was initiated 8.6 months after the initial SRS, and three courses were administered [17]. The patient’s neurological symptoms, which were mainly attributed to the local progression of the right thalamo-mesencephalic lesion, gradually improved after the third SRS. The two re-irradiated lesions showed favorable both clinical and radiographic responses (Figures 6A-6L, 7A-7L). The two newly manifested lesions of 0.66 cm^3^ and 0.40 cm^3^ showed complete responses (CRs) without subsequent progression during the 7.3-month imaging follow-up (Table 2 and Figures 8A-8N).

Eleven months after the initial SRS, a fourth 5-fr SRS was required to salvage two limited meningeal disseminations in the right occipital lobe near the resection cavity (data not shown). However, 12 months after the initial SRS, the patient was transferred to palliative care because of declining performance status. Although non-CE MRI 15.7 months after the initial SRS showed controlled intracranial metastases, continued analgesia and sedation for cancer-related pain were required. The patient died 18.8 months after the initial SRS.

The serial MRIs that were observed for more than one year after SRS were retrospectively reviewed for evaluating the dose-response relationships in the nine small BMs of ≤0.25 cm^3^ (Table 2). Only one of nine lesions had poor local control: the right frontal lesion (0.13 cm^3^) treated with 8-fr SRS resulted in PR followed by smoldering, in which the GTV minimum dose (D_min_) was 48.9 Gy (BED_10_ 78.8 Gy) with 91.2% IDS, and the GTV coverage with 49.6 Gy was 93% (Figures 8A-8N). The other two smaller lesions resulted in CRs, in which the GTV D_min_ was 49.0 Gy (BED_10_ 79.0 Gy) with 91.8% IDS (Table 2). Meanwhile, all six BMs (0.01-0.25 cm^3^) treated with 3-fr SRS showed sustained tumor regression, in which the GTV D_min_ was ≥35.0 Gy (BED_10_ 75.8 Gy) with ≥88.9% IDS. In the four lesions with local progression after 8-fr SRS, the duration from SRS to radiological local progression was associated with a GTV coverage value of 49.6 Gy. Thus, the durations with the GTV coverage of 70%, 93%, 97%, and 98% were 2.8 (Figures 3A-3L), 8.2 (Figures 8A-8N), 4.5 (Figures 1A-1L), and 8.2 months (Figures 2A-2L), respectively. Hence, a lower GTV coverage with 49.6 Gy tended to shorten the time to local progression, especially for >0.7 cm^3^ BMs.

As BEDs and corresponding absolute doses vary depending on the model formula and an alpha/beta ratio, the difference and variation in the BEDs and corresponding multi-fraction physical doses that are equivalent to a single fraction of 24 Gy are shown in Table 4. A single fraction of 24 Gy generally yields approximately 95% one-year local tumor control probability [1,7,14].

**Table 4:** Difference and variation of biologically effective doses and corresponding absolute doses in eight, five, and three fractions, for estimating the dose equivalent to a single fraction of 24 Gy as functions of the model formula and an alpha/beta ratio. LQ: linear-quadratic; LQC: linear-quadratic-cubic; LQL: linear-quadratic-linear; BED: biologically effective dose; fr: fraction

Model formula	LQ	LQ	LQ	LQC	LQC	LQL
Alpha/beta ratio	10	12	20	10	12	10
BED	81.6 Gy	72.0 Gy	52.8 Gy	56.0 Gy	50.7 Gy	48.4 Gy
Absolute dose (8 fr)	50.2 Gy	48.0 Gy	41.9 Gy	38.8 Gy	37.4 Gy	35.5 Gy
Absolute dose (5 fr)	43.6 Gy	42.3 Gy	38.2 Gy	34.9 Gy	33.9 Gy	32.5 Gy
Absolute dose (3 fr)	36.7 Gy	36.0 Gy	33.8 Gy	30.6 Gy	30.1 Gy	29.3 Gy

## Discussion

Despite the relatively high baseline prescription dose of 49.6 Gy (BED_10_ 80 Gy) to the GTV, in addition to mostly sufficient coverage of 40 Gy (BED_10_ 60 Gy) to 2 mm outside the GTV boundary, the initial 8-fr SRS resulted in local control failures in the four lesions (67%), including even a small lesion of 0.13 cm^3^. As mentioned above, the GTV coverage with 49.6 Gy was <98%, except in one lesion, and the D_max_ was ≤57 Gy (BED_10_ <100 Gy). The relatively homogeneous GTV doses resulted in no obvious tumor shrinkage during 8-fr SRS, whereas partial cystic enlargement and tumor displacement relative to the cranium inevitably led to dose falloff in parts of each tumor boundary [8,12,16]. In addition, a lower GTV coverage with 49.6 Gy shortened the time to local progression. Meanwhile, the two lesions of <0.1 cm^3^ with a D_min_ of ≥49 Gy were well controlled. The difference in local control may be attributed to differences in tumor cell numbers and internal radioresistance compared to those in larger lesions. Given that GTV coverage with ≤80% IDS of 53 Gy in 10 fr (BED_10_ 81 Gy) can improve local control for BMs >10 cm^3^, the local control failures in 8-fr SRS were mainly attributed to insufficient doses to both the margin and interior of the GTVs [5]. In addition to adequate coverage of a GTV boundary with 49.6 Gy, steep dose increase inside the GTV boundary with at least <80% IDS coverage would be important for long-term local control in 8-fr SRS. The anti-tumor efficacy of ≥49.6 Gy in 8 fr may be similar to that of a single fr of 24 Gy with a one-year tumor control probability of 95% [1]. In this respect, among the various formulae and alpha/beta ratios, the linear-quadratic (LQ) model-based BED_10_ seems to be the most suitable for predicting similar anti-tumor efficacies of single- and 8-fr SRS (Table 4). Furthermore, to ensure BED_10_-based GTV dose heterogeneity equivalent to that of a single fraction of 24 Gy with 80% IDS covering (D_max_ 30 Gy, BED_10_ 120 Gy), ≤75% IDS covering with 49.6 Gy in 8 fr would be suitable (Figure 5B and Table 5).

**Table 5:** Guidelines for physical marginal doses of a gross tumor volume (GTV), the GTV dose inhomogeneities, and dose gradients inside and outside the GTV boundary, according to the number of fractions, to ensure certain biologically effective doses. *Three millimeter for large lesions (e.g., >10 cm^3^). **The %IDS for a BED_10_ of ≥50 Gy, relative to the D_max_. ***The %IDS for a BED_10_ of ≥80 Gy, relative to the D_max_. As the number of dose fractions increases, the GTV boundary and 2-3 mm outside the GTV need to be covered with lower %IDSs. BED_10_: biologically effective dose based on the linear-quadratic formula with an alpha/beta ratio of 10; fr: fraction; IDS: isodose surface; D_max_: maximum dose; GTVs: gross tumor volumes

Variables		1 fr	3 fr	5 fr	8 fr	10 fr
GTV + 2 (-3*) mm	BED_10_ ≥50 Gy	≥18.0 Gy	≥27.3 Gy	≥30.9 Gy	≥34.9 Gy	≥36.6 Gy
	%IDS**	≤60.0%	≤58.2%	≤54.8%	≤53.0%	≤51.9%
GTV boundary	BED_10_ ≥80 Gy	≥23.8 Gy	≥36.3 Gy	≥43.1 Gy	≥49.6 Gy	≥52.5 Gy
	%IDS***	≤79.3%	≤77.4%	≤76.4%	≤75.3%	≤74.5%
	BED_10_ ≥81.6 Gy	≥24.0 Gy	≥36.7 Gy	≥43.6 Gy	≥50.2 Gy	≥53.3 Gy
GTV - 2 mm	BED_10_ ≥100 Gy	≥27.1 Gy	≥46.4 Gy	≥50.0 Gy	≥58.0 Gy	≥61.9 Gy
GTV center or D_max_	BED_10_ ≥120 Gy	≥30.0 Gy	≥46.9 Gy	≥56.4 Gy	≥65.9 Gy	≥70.5 Gy

The BMs of ≤0.25 cm^3^ treated with ≥89% IDS to the D_min_ of ≥35 Gy in 3 fr (BED_10_ ≥76 Gy) were well controlled (Table 2). Thus, for small lesions, a 3-fr dose based on BED_12_ or BED_10_ may provide an efficacy similar to that of a single fraction of 24 Gy (Table 4). The BMs of 0.40 cm^3^ and 0.66 cm^3^ with ≤77% IDS to the D_min_ of ≥43 Gy in 5 fr (BED_10_ ≥80 Gy) were also well controlled (Table 2 and Figures 8A-8N). The maximum responses of CRs may be enhanced with chemoimmunotherapy initiated immediately after the completion of 5-fr SRS. Taken together, the elementary LQ model-based BED_10_ may be the most suitable for estimating the anti-BM efficacies of 3-, 5-, and 8-fr SRS doses, which are similar to those of a single fraction of 24 Gy. GTV coverage with a BED_10_ of ≥80 Gy in 1-10 fr generally seems to be important for ensuring excellent local control. Furthermore, a BED_10_ of ≥81.6 Gy may be suitable to rigorously ensure similar efficacy to a single fraction of 24 Gy (Table 5). If consistent GTV coverage using a BED_10_ of 80 Gy with 80% IDS is used, regardless of the number of dose fractions, the BED_10_ in the GTV center decreases as the number of fractions increases (Table 5). Therefore, the larger the number of dose fractions, the more inhomogeneous the GTV dose should be designed (Table 5).

In the present case, we had to manage the challenging issue of re-irradiation for recurrences after 8-fr SRS with the BED_10_ of 80 Gy to the GTVs. To render re-irradiation clinically beneficial, sufficient alleviation of the relevant mass effect, that is, early and sufficient tumor shrinkage, is required; nevertheless, palliative and conservative doses are commonly used for re-irradiation with SRS [9]. Although the imaging and clinical follow-up periods after re-SRS were limited to 7.3 and 10.4 months, respectively, the 5-fr re-SRS with sufficient GTV coverage of 43 Gy and ≤68% IDS covering resulted in favorable responses both clinically and radiologically. Eight fractions of the initial SRS may render the surrounding brain immune to a significant ARE after re-SRS with the high BED_10_. Bevacizumab, which was included in chemoimmunotherapy, could also attenuate AREs; however, mild radiation-induced edema appeared only in the right frontal lobe after discontinuation (Figures 6A-6L, 7A-7L) [18]. Taken together, the criteria for determining the dominance of tumor regrowth and indication for re-SRS may include the following: insufficient GTV coverage (<98%) with a BED_10_ of 80 Gy, relatively homogeneous GTV doses, and dose/fractionation selection with adequate consideration of brain tolerance and volume effects in prior mfSRS, in addition to progression with T1/T2 matching.

Appropriate design and implementation of an initial SRS are important and should be prioritized to avoid re-treatment. Throughout the course of the present case, we renewed our recognition of the following: maximum response of PR indicates a residual viable tumor that will eventually regrow unless controlled with anti-cancer pharmacotherapy; thus, PR, especially within several months after SRS, does not guarantee excellent treatment [8]. Aside from LAC harboring major genetic alterations, for which low-dose SRS and molecular-targeted drugs are effective [19,20], complete tumor necrosis, including potential microscopic brain infiltration, should be aimed at achieving long-term sustained tumor control, especially in squamous cell carcinoma and pan-negative LAC, similar to the present case [8].

In isolated and symptomatic BMs, early symptom relief is necessary, and SRS is frequently initiated without determination of anti-cancer pharmacotherapy or its concurrent use, as in the present case. Subsequent anti-cancer medications can enhance anti-BM efficacies and mitigate potential AREs. The significance of anti-cancer pharmacotherapy and its appropriate selection after SRS of isolated BMs from pan-negative LAC remain issues for future investigation. In addition, the brain tolerance for 8-10 fr SRS, that is, dose-volume parameters relevant to symptomatic brain radionecrosis, remains a subject for further investigation [5].

## Conclusions

Sufficient GTV coverage with a BED of ≥80 Gy and steep dose increase inside the GTV boundary (extremely inhomogeneous GTV dose) are important in 8-fr SRS for ensuring excellent local control of BMs from pan-negative LAC. For local progression following mfSRS that does not fulfill both criteria, re-SRS with the above planning scheme can be an efficacious and safe treatment option for at least six months, especially in cases in which the prior SRS was performed with dose/fractionation selection under adequate consideration of brain tolerance. The elementary BED_10_ seems to be the most suitable for estimating the anti-tumor efficacies of SRS doses in 3-8 fr, similar to that of a single fraction of 24 Gy.
